# Hyperphosphatemia and hs-CRP Initiate the Coronary Artery Calcification in Peritoneal Dialysis Patients

**DOI:** 10.1155/2017/2520510

**Published:** 2017-02-22

**Authors:** Da Shang, Qionghong Xie, Bin Shang, Min Zhang, Li You, Chuan-Ming Hao, Tongying Zhu

**Affiliations:** ^1^Division of Nephrology, Huashan Hospital, Fudan University, Shanghai 200040, China; ^2^Division of Nephrology, Dezhou People's Hospital, Shandong 253014, China

## Abstract

*Background.* Coronary artery calcification (CAC) contributes to high risk of cardiocerebrovascular diseases in dialysis patients. However, the risk factors for CAC initiation in peritoneal dialysis (PD) patients are not known clearly.* Methods.* Adult patients with baseline CaCS = 0 and who were followed up for at least 3 years or until the conversion from absent to any measurable CAC detected were included in this observational cohort study. Binary logistic regression was performed to identify the risk factors for CAC initiation in PD patients.* Results.* 70 patients recruited to our study were split into a noninitiation group (*n* = 37) and an initiation group (*n* = 33) according to the conversion of any measurable CAC during their follow-up or not. In univariate analysis, systolic blood pressure, serum phosphorus, fibrinogen, hs-CRP, serum creatinine, and triglycerides were positively associated with the initiation of CAC, while the high density lipoprotein and nPCR did the opposite function. Multivariate analysis revealed that hyperphosphatemia and hs-CRP were the independent risk factors for CAC initiation after adjustments.* Conclusions.* Hyperphosphatemia and hs-CRP were the independent risk factors for CAC initiation in PD patients. These results suggested potential clinical strategies to prevent the initiation of CAC in PD patients.

## 1. Introduction

Cardiovascular events (CVEs) are the leading cause of death in patients on peritoneal dialysis (PD). Traditional risk factors like hypertension, diabetes, hyperlipideamia, and male gender cannot explain the abnormally high incidence of CVD in ESRD patients [1, 2]. Compared to the general population, the chronic kidney disease (CKD) and dialysis patients were characterized by mineral metabolism disorder, oxidative stress, and a poor nutritional state, resulting in more prevalent and markedly more severe vascular calcification.

Vascular calcification can occur in the arterial intimal layer in association with atherosclerosis, or in the arterial medial layer independent of atherosclerotic disease. Recently, though none of the methods can reliably distinguish between atherosclerotic and medial calcification, vascular calcification, especially the coronary artery calcification score (CaCS) assessed by the computerized tomography [3], has been reported to be an independent predictor of all-cause mortality and CVEs in CKD and dialysis patients [4–6]. A major area of interest concerns the reasons behind the development and accelerated progression of CaCS in patients with ESRD. In vitro and in vivo, HDL inhibits the osteogenic differentiation pathway [7], while phosphate will induce arterial calcification in a dose-dependent manner, which is also associated with the upregulation of proteins involved in bone formation and the phenotypic differentiation [8, 9]. In dialysis patients, age, hypercalcemia, hyperphosphatemia, PD duration, hyperlipidemia, and inflammation are considered to be related to the CAC progression [10–16].

More interested, the conversion from no CAC to any CAC reflects an important step of the disease process. In general population with zero CAC at baseline, revealing age, LDL cholesterol, systolic blood pressure, and current smoking were independent predictors of CAC onset [16, 17]. However, there were few clinical studies about the risk factors to initiate the CAC in PD patients. Accordingly, we performed a prospective study of 70 patients with zero CAC at baseline in order to identify the initiator of CAC in PD patients.

## 2. Methods and Materials

### 2.1. Study Population

Adult PD patients treated at division of nephrology, Huashan Hospital Fudan University in China from June 2004 to march 2013 were recruited in this observational cohort study. They received regular PD treatment and underwent a series of coronary artery calcification score (CaCS) measurements by MSCT at baseline and semiannual or annual repeat scans during the follow-up. The patients with baseline CaCS = 0 and were followed up for at least 3 years or until the conversion from absent to any measurable CAC were eligible for the present analysis. The demographic characteristics, laboratory test data, and adequacy of PD were collected. Binary logistic regression was performed to identify the initiators of CAC in PD patients.

Patients were excluded: the CAC at baseline were not zero; the follow-up time of patients with the CAC remaining at zero were less than 3 years due to various reasons. All the participants provided their written informed consent, and the protocol of the study was approved by the ethics committee of Huashan Hospital at Fudan University.

### 2.2. Data Collection and Statistical Analysis

The recruited PD patients were divided into two groups, noninitiation and initiation group, according to their follow-up of whether CaCS remained at zero or not. The CaCS was recorded as just as we previous described [6, 14]. Demographic characteristics and comorbidities (diabetes mellitus, hypertension, and CVD) were recorded at baseline. Laboratory measurements, such as calcium-phosphate metabolism, lipids and inflammation markers, were collected every 3 months. PD adequacy was evaluated every 6 months using Baxter PD Adequest 2.0 software (Baxter Healthcare Corporation, Deerfield, IL, USA). The average values of these indexes during the total follow-up time in noninitiation group were calculated, while in initiation group the average was acquired within one year before any measurable CAC.

Statistical analysis of the data was performed using SPSS software, version 17.0. The variables expression and the statistical test were described in our previous study [14]. Binary logistic regression was used to identify the independent risk factors for CAC initiation in PD patient.

## 3. Results

### 3.1. Patients' Characteristics

Of 550 PD patients in our PD center, 112 (21.33%) did not show any CaCS at the baseline. But as of March 2016, among these patients, only 70 patients (54.4 ± 13.4 years old, 36 men) whose baseline CaCS = 0, with a follow-up longer than 36 months or any detected CAC, were considered for the analysis. The dialysate volume was 6.8 ± 1.4 L every day and the long and short dwelling times were 12 hours and 4 to 6 hours, respectively. All patients used the Dianeal PD4 (1.25 mmol/L calcium, 2 L) and Dianeal PD2 (1.75 mmol/L calcium, 2 L) from Baxter Healthcare Corporation. No icodextrin was used because it is not available in China.

**Figure 1:**
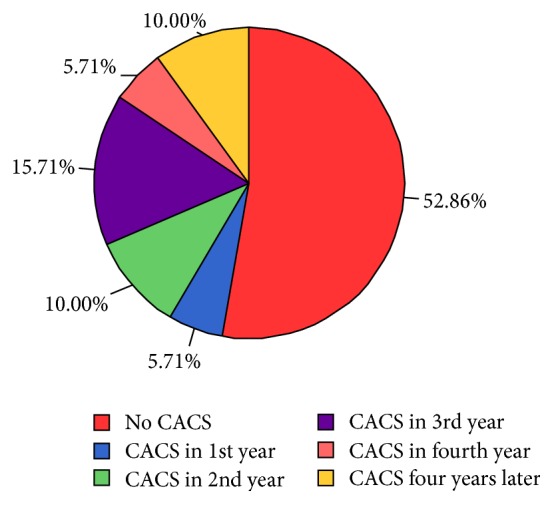
CACS-initiated time period in PD patients.

**Figure 2:**
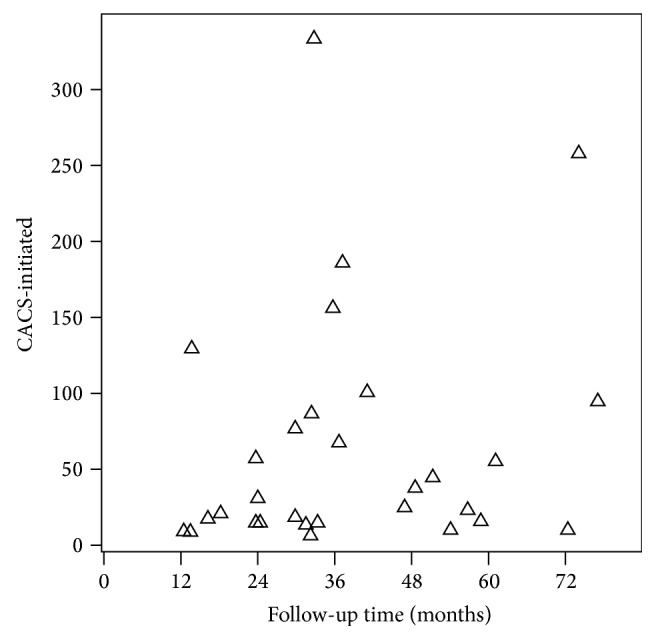
CACS when first detected in initiated group.

Among the 70 patients with a mean follow-up of 54.0 ± 16.7 months, 4 patients (5.71%) showed the CaCS in the first follow-up year, 7 (10%) in the second year, 11 (15.71%) in the third year, 4 (5.71%) in the fourth year, and 7 (10%) in later until March 2016, and they were grouped into the initiation group, and the other 37 (52.86%) remaining at zero CaCS during the follow-up time were grouped into the noninitiation group (Figure 1). And in the initiation group, the initiation CaCS, which was defined as the first detected quantitative CaCS without zero (CACS-initiated), was 29.6 (16.6, 100.7). Among these patients, 10 (30.3%) had the CaCS-initiated more than 100 and 19 (57.6%) less than 50 (Figure 2).

The follow-up time of initiation group (19 men, 57.6%) was 55.9 ± 16.2 months; similarly the noninitiation group (17 men, 45.9%) was 52.2 ± 17.2 months. There was weak difference in the history of smoking (9/37 versus 14/33, *p* = 0.116) and BMI (*p* = 0.087) between the two groups. The systolic blood pressure (SBP) was higher in the initiation group (*p* = 0.030), while the diastolic blood pressure (DBP) was not (*p* = 0.322). There were no significant differences in the baseline duration of PD therapy between the groups (*p* = 0.638). The glucose reabsorption (*p* = 0.144) and the level of 25-OH-VitD (*p* = 0.59) between the groups were also similar. Sixty-six patients (94.3%) received continuous ambulatory peritoneal dialysis, while others received daytime ambulatory peritoneal dialysis. During the follow-up period, 6 patients died (1 in noninitiation group versus 5 in initiation group, *p* = 0.065) and 11 experienced CVEs (3 in noninitiation group versus 8 in initiation group, *p* = 0.066) (Tables 1 and 2).

In total, 45 (64.3%) patients had an average residual renal Ccr of >20 L/week and 46 (65.7%) had an average total Ccr of >60 L/week. Twenty-seven (38.6%) patients had a serum phosphate level of >5.5 mg/dL, and 52 (74.3%) had a level of >4.5 mg/dL. Among the included patients, 51 patients (72.9%) received calcium carbonate treatment (29/37 versus 23/33; *p* = 0.577) and 49 received calcitrol treatment (31/37 versus 18/33; *p* = 0.008). Else, twenty-three (62.2%) patients take the lower-lipid agent to modify the lipids in noninitiation group and fourteen (42.4%) in initiation group (23/37 versus 14/33, *p* = 0.101) (Tables 1 and 2).

### 3.2. Hyperphosphatemia and hs-CRP as Independent Risk Factors for CAC Initiation

The two groups were weakly different in BMI (*p* = 0.087), uric acid (UA) (*p* = 0.087), Homa-IR (*p* = 0.105), and residual CCR (*p* = 0.056), while they were similar in hemoglobin, AKP, ferritin, cholesterol, LDL, iPTH, and adjusted calcium. The univariate analysis showed that SBP (*p* = 0.030), hs-CRP (*p* = 0.005), fibrinogen (*p* = 0.019), serum creatinine (*p* = 0.011), triglycerides (*p* = 0.027), and serum phosphorus (*p* < 0.001) were the potential risk factors for CAC initiation in PD patients, while the HDL (*p* < 0.001) and nPCR (*p* = 0.031) may prevent the PD patients from suffering the CAC (Tables 1 and 2).

Multivariate analysis using logistic regression forward conditional method revealed that serum phosphate level and hs-CRP were independent risk factors for CAC initiation after adjusting for gender, BMI, SBP, fibrinogen, serum creatinine, UA, nPCR, triglycerides, HDL, Homa-IR, and adjusted residual CCR (Table 3).

## 4. Discussion

Previous study revealed that the baseline CAC was associated with the progression of CAC in general and dialysis patients [14, 16]. We sought to identify risk factors that determined incident CAC > 0 in PD patients with the hope of potential treatment preventing the vascular calcification from the very early status. In our study, we found serum phosphate level and hs-CRP were independent risk factors for CAC initiation in PD patients. These results suggested potential strategies to prevent the initiation of CAC in PD patients.

In 1261 general patients with zero CAC at baseline, age, LDL, SBP, and current smoking were independent predictors of CAC onset after 5.1 years of follow-up [17]. Results from the Multi-Ethnic Study of Atherosclerosis (MESA) also showed the age, SBP, smoking, LDL, HDL, and creatinine were related to the incident CAC risk [18, 19]. In our study, the SBP in noninitiation group were controlled better than that in initiation group, which reveal the role of blood pressure in initiating the CAC in PD patients. Calcified arteries had upregulation of angiotensin 1 receptor and treatment with an angiotensin receptor blocker prevented the calcification [20]. However, in a CKD model, treatment with enalapril had no effect on vascular calcification in a CKD model [21]. The hypertension was not a common risk factor for CAC, mainly because patients with CAC have hypertension as a manifestation in vascular calcification patients, which may not be so to patients without CAC.

As we know, HDL reverse the atherosclerosis and reduce the risk of CVD due to reversing the cholesterol transport to liver, inhibition of oxidation, and inflammation [22]. Recently, Farhad Parhami demonstrates the ability of HDL to inhibit the calcification of vascular cells [7], and HDL benefits the arterial wall by affecting the endothelia BMP-signaling, essential for endothelial cell survival and prevention of vascular calcification, respectively [23]. However, we failed the correlation in multivariate analysis may be due to the impaired HDL antioxidant and anti-inflammatory properties in ESRD patients to some degree [24].

In general population, compared with no detectable CAC, people with CAC had significantly higher BMI, SBP, history of smoking, and fibrinogen [25]; data from MESA also showed fibrinogen was associated with CAC presence and burden [26]. As we know, fibrinogen is not only associated with the enhanced blood viscosity as a main coagulation protein, but also involved in the atherosclerosis [27]. Thus, the control of the fibrinogen may be a potential therapy in preventing from CAC in PD patients.

Vascular calcification is associated with additional nontraditional factors that may be unique to CKD, such as disordered mineral metabolism. In previous studies, the phosphate was proved to be associated with CAC progression and the mortality in both general and CKD patients [14, 28–31]. In our study, the hyperphosphatemia was related to the CAC initiation in PD patients. The mechanisms by which phosphate induces the CAC in CKD may include promoting the osteochondrogenic phenotype change of vascular smooth muscle cells (VSMC) [32, 33], phosphate-induced apoptosis of VSMC [34–36], the inhibition of osteoclast differentiation [37], and phosphorus-mediated elevation of FGF-23 [38, 39]. In El-Abbadi's uremic models, the high phosphate fed mice were prior to mineralization, consistent with the role as an initiating event in SMC phenotype change and calcification [9]. Hyperphosphatemia contributes to several mechanisms that initiate or advance the progression of vascular calcification and is emerging as a key regulator of calcification in patients with kidney disease [40] and supplies a potential strategy to prevent the CAC to any measurable degree as cardiovascular risk increases continuously even at very low degrees of CAC burden.

In addition, inflammation may promote vascular calcification by releasing “tumor necrosis factor *α*,” which triggers the Wnt signaling pathway, resulting in osteogenic differentiation of VSMCs [41, 42]. Our study shows the inflammation factor, hs-CRP, is higher in the initiation group, as determined by the multivariate analysis. This finding may also be supported by Aikawa's study. Osteogenesis is associated with local inflammation and macrophage infiltration in atherosclerosis and atherosclerotic mineralization is linked with inflammation at the earliest stages [43].

The limitation to our study was that it was an observational, single-center, and relatively small study. Though we concluded that hyperphosphatemia and hs-CRP initiate the CAC in PD patients, we did not examine whether the control of the related factor could prevent the CAC initiation.

## 5. Conclusion

In summary, these findings suggest hyperphosphatemia and hs-CRP are the independent risk factors of CAC initiation in PD patients, supplying potential strategies to prevent the CAC to any measurable degree at the very early status.

## Figures and Tables

**Table 1:** Clinical characteristics of the peritoneal dialysis patients.

	Coronary artery calcification			*p* value
	Noninitiation (*n* = 37)	Initiation (*n* = 33)	Total (*n* = 70)	
Age (years)	53.9 ± 12.0	54.9 ± 15.1	54.4 ± 13.4	0.749
Gender (male)	45.9%	57.6%	51.40%	0.335
BMI (kg/m^2^)	22.5 ± 3.6	24.0 ± 3.5	23.2 ± 3.6	0.087
Smoking	23.5%	41.9%	32.30%	0.116
Cause of ESRD				0.241
Glomerulonephritis	89.2%	78.8%	84.30%	
Diabetes mellitus	8.1%	15.2%	11.40%	
Others	2.7%	6.1%	4.30%	
SBP (mmHg)	125 ± 20	136 ± 21	130 ± 21	0.030
DBP (mmHg)	80 ± 11	82 ± 12	81 ± 12	0.322
CAPD	91.9%	93.9%	92.90%	0.742
CVD	8.1%	24.2%	15.70%	0.066
All-cause deaths	2.7%	15.2%	8.60%	0.065
Follow-up time (months)	52.2 ± 17.2	55.9 ± 16.2	54.0 ± 16.7	0.351
Baseline duration of PD (months)	0.2 (−0.4,2.6)	0.13 (−0.3,6.3)	0.2 (−0.3,4.3)	0.638
CACS-initiated	0 (0, 0)	29.6 (16.6, 100.7)	0.9 (0, 25.9)	<0.001
Calcitrol	83.8%	54.5%	70.0%	0.008
Statin	62.2%	42.4%	52.9%	0.101
Calcium carbonate	75.7%	69.7%	72.90%	0.577

BMI: body mass index; SBP: systolic blood pressure; DBP: diastolic blood pressure; CAPD: continuous ambulatory peritoneal dialysis; CVD: cardiovascular disease; CACS-initiated: the CACS we detected firstly when coronary artery calcification occurred.

**Table 2:** Laboratory characteristics of the peritoneal dialysis patients.

	Coronary artery calcification			*p* value
	Noninitiation (*n* = 37)	Initiation (*n* = 33)	Total (*n* = 70)	
Hemoglobin (g/L)	106.0 ± 10.1	103.6 ± 13.4	104.9 ± 11.8	0.398
iPTH (ng/dL)	347 ± 153	441 ± 324	391 ± 251	0.121
Adjusted calcium (mg/dL)	10.3 ± 1.2	10.2 ± 0.9	10.2 ± 1.0	0.633
Phosphorus (mg/dL)	4.62 ± 0.79	5.90 ± 1.33	5.22 ± 1.25	<0.001
Transferrin (g/L)	1.84 ± 0.29	1.81 ± 0.24	1.83 ± 0.27	0.615
Ferritin (ug/L)	259 ± 88.8	278 ± 143	268 ± 117	0.511
Fibrinogen (g/L)	3.93 ± 0.85	4.43 ± 0.90	4.17 ± 0.90	0.019
Pro-BNP (pg/mL)	1987 (758, 5591)	2298 (784, 11154)	2191 (767, 8755)	0.362
Serum creatinine (umol/L)	851 ± 285	1065 ± 399	952 ± 357	0.011
BUN (mmol/L)	21.1 ± 5.1	23.1 ± 14.3	22.0 ± 10.5	0.428
UA (mmol/L)	0.43 ± 0.07	0.46 ± 0.09	0.45 ± 0.08	0.087
AKP (U/L)	84.8 ± 24.9	90.7 ± 59.9	87.6 ± 44.7	0.586
Albumin (g/L)	36.2 ± 4.1	36.2 ± 3.2	36.2 ± 3.7	0.944
nPCR (g/kg*∗*d)	0.96 ± 0.19	0.87 ± 0.12	0.92 ± 0.16	0.031
hs-CRP(mg/dL)	1.75 ± 2.14	4.37 ± 5.0	2.98 ± 3.96	0.005
HbA1C	5.52 ± 0.65	5.83 ± 0.75	5.67 ± 0.71	0.070
Homa-IR	2.85 ± 1.96	4.12 ± 4.22	3.45 ± 3.26	0.105
Cholesterol (mg/dL)	179 ± 31	179 ± 40	179 ± 35	0.987
Triglycerides (mg/dL)	167 ± 108	261 ± 227	211 ± 180	0.027
LDL (mg/dL)	90.4 ± 22.8	86.7 ± 23.1	88.7 ± 22.8	0.499
HDL (mg/dL)	44.4 ± 9.7	34.8 ± 9.8	39.9 ± 10.8	<0.001
Lipidprotein A (mg/dL)	250 ± 204	243 ± 173	247 ± 189	0.879
25-OH-VitD (nmol/L)	36.8 ± 9.6	38.4 ± 10.1	37.7 ± 9.8	0.59
Glucose reabsorption (g/W/m^2^)	363 ± 116	404 ± 113	382 ± 116	0.144
Adjusted total ccr (L/W)	71.7 ± 20.0	63.1 ± 16.6	67.7 ± 18.8	0.057
Adjusted PD ccr (L/W)	37.9 ± 8.0	39.8 ± 6.9	38.8 ± 7.5	0.313
Adjusted residual CCR (L/W)	33.8 ± 25.1	23.4 ± 18.8	28.9 ± 22.8	0.056

iPTH: intact parathyroid hormone; pro-BNP: pro-B-type natriuretic peptide; BUN: blood urea nitrogen; UA: uric acid; AKP: alkaline phosphatase; nPCR: normalized protein catabolic rate; hs-CRP: high-sensitivity C-reactive protein; Ccr: creatinine clearance rate; LDL: low-density lipoprotein; HDL: high-density lipoprotein; Lp (a): lipoprotein (a); glucose reabsorption: the glucose reabsorption per week adjusted by body surface area.

**Table 3:** Multivariate analyses of the selected possible risk factors for CaCS initiation in PD patients.

		OR	*p* value
Step 1	Phosphorus (mg/dL)	3.312 (1.755, 6.248)	<0.001
Step 2	hs-CRP (mg/dL)	1.528 (1.130, 2.067)	0.006
	Phosphorus (mg/dL)	4.844 (2.190, 10.715)	<0.001

Variables analyzed by logistic regression forward conditional: gender, BMI, SBP, phosphorus, fibrinogen, serum creatinine, UA, nPCR, hsCRP, triglycerides, HDL, Homa-IR, and adjusted residual CCR.
